# Review of “orthopaedic biomechanics” edited by Beth A. Winkelstein

**DOI:** 10.1186/1475-925X-12-34

**Published:** 2013-04-18

**Authors:** Solomon P Samuel, Nathan C Tiedeken

**Affiliations:** 1 Department of Orthopaedic Surgery, Einstein Medical Center, Philadelphia, PA 19141, USA

**Keywords:** Biomechanics, Finite element analysis, Injury, Gait analysis, Orthopedics, Bone biomechanics

## Abstract

This article is a review of the book “orthopaedic biomechanics” edited by Beth A. Winkelstein. This book (hardcover) was published by CRC Press, Taylor & Francis Group, FL in 2012. The contents of the book and its relevance to orthopedic research or practice is discussed in this invited review.

## Book details

Orthopaedic Biomechanics CRC Press, FL; Winkelstein, BA; 2012; 639 Pages. List price: USD 179.95, ISBN 9781439860939 (Hardback)

“Orthopaedic Biomechanics” was released in 2012 and edited by Beth A. Winkelstein, PhD. The list price of the book ($179.95) is comparable with similar books published in this field. This text is intended for both the novice and established researcher working the field of orthopaedic biomechanics. However, this book assumes that the reader has some basic knowledge about biomechanics, finite element modeling, and biomaterials used in orthopedic surgery. Composed of twenty-one chapters with approximately 3,000 references, “Orthopaedic Biomechanics” provides an exhaustive literature review of the most current published data in orthopaedic biomechanical engineering. This work focuses on the vast and complex nature of the field of orthopedic biomechanics that challenges both researchers and clinicians. The book is divided into three sections that begin with basic concepts, principles, and function of tissues. Each section builds upon the previous and culminates with the integration of biomechanical properties and their application in a clinical setting. “Orthopaedic Biomechanics” is an illustrated text including diagrams, anatomical dissections, experimental charts, finite element equations, and color photographs. Each chapter begins with an introductory overview and reviews the pertinent micro and macro-anatomy before discussing the chapter topic. A brief summary incorporating future research considerations emphasize the most important topics and leave the reader with a complete understanding of the chapter.

Written by some of the leaders in their respective fields, “Orthopaedic Biomechanics” discusses current trends, terminology and data with regard to well established principles in orthopaedic biomechanics. It also confronts contemporary biomechanical topics like gender, developmental biomechanics, functional tissue engineering, prosthetics, and aging. These are subjects that are receiving much attention in both clinical and research settings. This book also provides a review of all major orthopaedic injuries, including but not limited to, the skull, chest, spine, hand, and lower extremity. Biomechanical analysis of these injuries are extensively covered and well supported with informative vector diagrams and three dimensional computational imaging which further help simplify a complex topic. “Orthopaedic Biomechanics” recognizes the importance of not only updating the reader on well known biomechanical orthopaedics, but also advancing the science by highlighting the expansion and future research on the latest subject matter.

In the following paragraph, we highlight several chapters which we believe are unique in “Orthopaedic Biomechanics.” Chapter five provides an extensive review on skull development. Basic biomechanical concepts of the skull and the effects of skull injury and healing on these properties are reviewed. Understanding and developing skull biomechanics is essential for improving protective devices in the sports and automotive industries. Temporomandibular joint (TMJ) mechanics are presented in chapter six. This chapter simplifies the very complex biomechanical environment of the jaw. Osseous, muscular, ligamentous, and cartilaginous structures on the jaw are explained and the current concepts of jaw biomechanics are reviewed. Chapters 7–10 involve normal and pathologic states of the spine, shoulder, hand and lower extremity and the biomechanics of each condition. Musculoskeletal development, gender, and aging, and the changes of each on the biomechanical properties are described in chapters twelve and thirteen. Understanding musculoskeletal development, differences in gender, and the effect of aging are essential for injury prevention in sports, orthopaedic implants and geriatric injury. These concepts are the frontier in the field of orthopaedic biomechanics. Chapter fourteen examines the merger of biomechanical properties with computational modeling. Pathology of injury to bone and joint structures are examined with the use of finite element modeling to effectively guide experimental designs. Chapter fifteen evaluates gait mechanics and the current technologies utilized in assessing normal and pathological bipedal ambulation. This chapter also shows how the current technology of gait analysis has greatly improved but is rarely integrated in the clinical setting. Chapters sixteen and seventeen discuss sports and non sports related orthopedic injuries. Biomechanical analysis of the mechanism of injury as well as injury risk models for all areas of the body are evaluated. Chapter 19 summarizes the latest biopolymer technological advancements and their use in treating orthopaedic pathologies.

In summary, this book is an exceptional reference manuscript for scientists at any level of training in the area of orthopaedic biomechanical engineering. With a strong devotion to biomechanical principles and their orthopaedic applications, this book bridges the divide between the basic science research and its effect on clinical practice. Due to this strong clinical emphasis, physicians who commonly encounter orthopedic injuries would greatly benefit from referencing this book. However, the average physician’s encounter of complex equations and emphasis on mathematical modeling will be the limiting factor in their use of this book. Despite this, we believe “Orthopaedic Biomechanics” achieved the rare accomplishment of creating a relevant text for not only orthopaedic biomechanical engineers but also clinical physicians. We thank the editors for inviting us to write this review.

## Competing interests

The authors declare no competing interest with regards to this invited review.

## Authors’ contribution

The authors contributed equally to the manuscript. Both authors read and approved the final manuscript.

